# Identification of Quantitative Trait Loci Associated With Partial Resistance to Fusarium Root Rot and Wilt Caused by *Fusarium graminearum* in Field Pea

**DOI:** 10.3389/fpls.2021.784593

**Published:** 2022-01-20

**Authors:** Longfei Wu, Rudolph Fredua-Agyeman, Stephen E. Strelkov, Kan-Fa Chang, Sheau-Fang Hwang

**Affiliations:** Department of Agricultural, Food and Nutritional Science, University of Alberta, Edmonton, AB, Canada

**Keywords:** *Pisum sativum* L., recombinant inbred lines (RIL), conidia suspension, SNP and SSR markers, linkage map construction and QTL mapping

## Abstract

Fusarium root rot, caused by a complex of *Fusarium* spp., is a major disease of field pea (*Pisum sativum*). The development of genetic resistance is the most promising approach to manage the disease, but no pea germplasm has been identified that is completely resistant to root rot. The aim of this study was to detect quantitative trait loci (QTL) conferring partial resistance to root rot and wilting, caused by five fungal isolates representing *Fusarium solani*, *F. avenaceum*, *F. acuminatum*, *F. proliferatum*, and *F. graminearum*. Evaluation of the root rot-tolerant cultivar “00-2067” and susceptible cultivar “Reward” was carried out with the five species. There was a significant difference (*p* < 0.001) between the mean root rot values of the two cultivars inoculated with the *F. avenaceum* (F4A) and *F. graminearum* (FG2) isolates. Therefore, in the QTL study, the F_8_ recombinant inbred line (RIL) population derived from “Reward” × “00-2067” was inoculated in the greenhouse (4 ×) with only F4A and FG2. The parents and F_8_ population were genotyped using 13.2K single nucleotide polymorphisms (SNPs) and 222 simple sequence repeat (SSR) markers. A significant genotypic effect (*p* < 0.05) and high heritability (79% to 92.1%) were observed for disease severity, vigor, and plant height following inoculation with F4A and FG2. Significant correlation coefficients were detected among and within all traits. This suggested that a high proportion of the genetic variance was transmitted from the parents to the progeny. However, no significant QTL (LOD > 3) were detected for the RILs inoculated with F4A. In the case of the RILs inoculated with FG2, 5 QTL for root rot severity and 3 QTL each for vigor and plant height were detected. The most stable QTL for plant height (*Hgt-Ps3.1*) was detected on Chrom5/LGIII. The two most stable QTL for partial resistance to FG2, *Fg-Ps4.1*, and *Fg-Ps4.2* were located in a 15.1-cM and 11.2-cM genomic region, respectively, on Chrom4/LGIV. The most stable QTL for vigor (*Vig-Ps4.1*) was found in the same region. Twenty-five major and moderate effect digenic epistatic interactions were detected. The identified region on chrom4/LGIV could be important for resistance breeding and marker development.

## Introduction

Globally, root rot is estimated to cause yield reductions of 10–30% in pulse crops, but losses can be as high as 100% in crops with severe infections under ideal environmental conditions (Oyarzun, 1993; Schneider et al., 2001; Schwartz et al., 2005; Cichy et al., 2007). As such, root rot is one of the most devastating diseases of field pea and other pulse crops in Canada and worldwide (Hwang and Chang, 1989; Feng et al., 2010; Chatterton et al., 2015, 2019; Gossen et al., 2016; Chang et al., 2017; Safarieskandari et al., 2020; Wu et al., 2021). The causal organisms of the pea root rot complex (PRRC) are soil-borne fungal and fungal-like organisms that include *Fusarium* spp., *Aphanomyces euteiches*, *Pythium* spp., *Phytophthora* spp., *Rhizoctonia* spp., *Didymella* spp. (formerly *Mycosphaerella* spp.), and *Ascochyta* spp. (Fletcher et al., 1991; Kaiser, 1992; Hwang et al., 1994; Xue et al., 1998; Bailey et al., 2003; Chang et al., 2005, 2013, 2014, 2017; Tyler, 2007; Díaz Arias et al., 2011).

Given their abundance and wide host range, the vast majority of the PRRC organisms are *Fusarium* species, although these may exhibit variable virulence toward different hosts. The various species identified in the Canadian prairies include *F. solani, F. avenaceum, F. oxysporum, F. graminearum*, *F. culmorum, F. acuminatum, F. redolens*, *F. sambucinum var. coeruleum, F. equiseti, F. poae, F. sporotrichioides*, and *F. tabacinum* (Kraft and Pfleger, 2001; Fernandez, 2007; Fernandez et al., 2008; Feng et al., 2010; Chittem et al., 2015; Wu et al., 2017; Chang et al., 2018; Zitnick-Anderson et al., 2018). Among these, *F. avenaceum*, *F. solani*, and *F. oxysporum* were reported to be the primary species causing significant Fusarium root rot (FRR) in the major field pea cultivation regions in Canada and worldwide (Kraft, 1981; Kraft and Pfleger, 2001; Wille et al., 2020).

The *Fusarium graminearum* species complex (FGSC) includes the major pathogens causing Fusarium head blight (FHB) of wheat, barley, oats, and other small grain cereals (O’Donnell et al., 2008). On cereal hosts, FGSC produces various mycotoxins known as trichothecenes [e.g., deoxynivalenol (DON), nivalenol (NIV), zearalenone (ZEN), and fumonisin B1 (FB1)], which are detrimental to human and animal health when ingested (van der Lee et al., 2015). While *F. graminearum* mainly affects cereals, this pathogen has been isolated from field pea in Canada, the USA, and Lithuania (Feng et al., 2010; Chittem et al., 2015; Rasiukevičiūtė et al., 2019). Rasiukevičiūtė et al. (2019) reported that field pea was the non-cereal crop most susceptible to *F. graminearum* compared with faba bean, fodder beet, oilseed rape, potato, and sugar beet.

At present, there are no sources of complete resistance to PRRC in field pea. Furthermore, higher global temperatures and excessive soil moisture associated with climate change have led to the increased incidence and severity of many plant diseases (Chakraborty et al., 2000; Dorrance et al., 2003; Gautam et al., 2013; Elad and Pertot, 2014). While tillage was reported to be beneficial to the soil environment, it did not suppress the development of FRR in field pea (Bailey et al., 1992). Seedling data and depth were reported to affect FRR in lentil (Hwang et al., 2000), but not in field pea (Chang et al., 2013). Crop rotations longer than 4 years are recommended for the management of root rot, but these are not always practical (Hwang and Chang, 1989; Bainard et al., 2017). Fungicidal seed treatments were reported to increase emergence and reduce root rot severity in the early growth stages of pea (Xue et al., 2000; Wu et al., 2019), with Apron Maxx (fludioxonil, metalayxyl-M and S-isomer), prothioconazole, fluopyram, and penthiopyrad, suppressing FRR in greenhouse and field experiments (Avenot and Michailides, 2010; Chang et al., 2013). However, some fungicides can also affect Rhizobia, leading to reductions in nodulation and nitrogen fixation (Chang et al., 2013), and their use is not environmentally friendly.

Genetic resistance offers the most promising way to control FRR and wilt in pea. However, there is no complete resistance to FRR in field pea, and only a few studies have reported QTL associated with partial resistance to this disease (Feng et al., 2011; McPhee et al., 2012; Coyne et al., 2015, 2019). Coyne et al. (2015, 2019) identified the major QTL for partial resistance to *F. solani*, *Fsp-Ps2.1*, to be on LGII (Chromosome 6), while four minor QTL were found on LGIII, IV, VI, and VII (Chromosomes 5, 4, 1, and 7, respectively). These QTL explained 44.4–53.4% of the total variance for resistance (Coyne et al., 2019). McPhee et al. (2012) detected one major QTL on LGIV (Chromosome 4) and two minor QTL on LGIII (Chromosome 5) to be associated with partial resistance to *F. oxysporum* race 2. The major QTL identified by McPhee et al. (2012), *Fnw4.1*, explained 68–80% of the phenotypic variance. Feng et al. (2011) identified one QTL controlling resistance to *F. avenaceum* on LGVII (Chromosome 7) in a rough map generated with 14 SSRs. The QTL identified in most of these studies had very large confidence intervals due to the limited number of markers used. The low marker density makes it difficult to apply the identified markers in pea breeding.

On the Canadian prairies, cereals are grown in tight rotations with canola, while the cultivation of field pea and other pulses is increasing (Bekkering, 2013; Gill, 2018). Boom-and-bust-type cycles of root rot diseases were highly correlated with crop rotation practices (Govaerts et al., 2007; Su et al., 2021). Therefore, the order of cultivation of crops in a rotation is important. The increased incidence and severity of FRR in field pea make the study of the genetic resistance to different *Fusarium* spp. an important research objective.

Therefore, the objectives of this study were to: (1) evaluate the partially resistant pea cultivar “00-2067” for resistance to different *Fusarium* spp. recovered from surveys for root rot in Alberta, Canada; (2) map the QTL associated with partial resistance to FRR using a segregating recombinant inbred line (RIL) pea population genotyped by Wu et al. (2021) and the most virulent of the *Fusarium* isolates; and (3) determine the stability of the QTL, accounting for disease severity, vigor, and plant height.

## Materials and Methods

### Plant Materials

One-hundred thirty-five RILs used by Wu et al. (2021) for mapping the QTLs associated with partial resistance to Aphanomyces root rot were included in this study. In brief, the Aphanomyces root rot-resistant pea parent “00-2067” developed by Dr. J. Kraft and V. A. Coffman at the Irrigated Agriculture Research and Extension Center in Prosser, WA, United States (Conner et al., 2013; Wu et al., 2021), was used in genetic crosses with the susceptible parent “Reward” (Bing et al., 2006) to produce F_1_ plants, which were then used to develop an F_8_ RIL population (Supplementary Figure 1) by the single-seed descent (SSD) method (Brim, 1966).

### *Fusarium* Isolates

Five single-spore isolates (SSI), S4C (*F. solani*), F4A (*F. avenaceum*), F037 (*F. acuminatum*), F039 (*F. proliferatum*), and FG2 (*F. graminearum*), representing the *Fusarium* species most frequently recovered from symptomatic pea plants in root rot surveys in Alberta, were used to screen the parental cultivars “00-2067” and “Reward.” Briefly, to obtain the SSIs, surface-sterilized pieces of root tissue with disease lesions were placed on potato dextrose agar (PDA) and incubated at 25°C for 2–3 days and then transferred to the peptone-pentachloronitrobenzene (PCNB) medium for further selection. Mycelial tips of the fungal isolates were cut from selected colonies under a stereomicroscope (Zeiss Axio Scope A1, Carl Zeiss Canada Ltd., Canada), and the water agar (WA) procedure was used to obtain SSI (Zitnick-Anderson et al., 2020). The species designation of each of the SSIs was confirmed based on morphology and evaluation with the PCR primer sets ITS4/ITS5 and EF-1/EF-2, while isolate virulence was confirmed by fulfilling Koch’s postulates (Feng et al., 2010; Chen et al., 2014; Zhou et al., 2014; Wu et al., 2017; Chang et al., 2018; Zitnick-Anderson et al., 2018).

### Inoculum Production

Conidial suspensions of the five isolates were generated following Son et al. (2013). Pure cultures of each *Fusarium* spp. were grown in Petri dishes on PDA under darkness at room temperature for 4–6 weeks. Sterile-distilled water was added to each Petri dish, and the surface of each colony was gently scraped with a sterile inoculation needle to dislodge the spores (and hyphal fragments), with the resulting suspension decanted into 250-ml Erlenmeyer flasks, containing a 100-ml autoclaved CMC medium (1.5% carboxymethyl cellulose, 0.1% yeast extract, 0.05% MgSO_4_⋅7H_2_O, 0.1% NH_4_NO_3_, 0.1% KH_2_PO_4_, 100-ml H_2_O). The flasks were covered in aluminum foil to block light and incubated on a rotary shaker at room temperature for 2 weeks. The suspension was centrifuged to collect conidia. The concentration of conidia was estimated with a hemocytometer and adjusted to a final concentration of 2 × 10^6^ spores ml^–1^ with sterile-deionized water.

### Screening of Recombinant Inbred Line Parents With Five *Fusarium* Species

**FIGURE 1:**
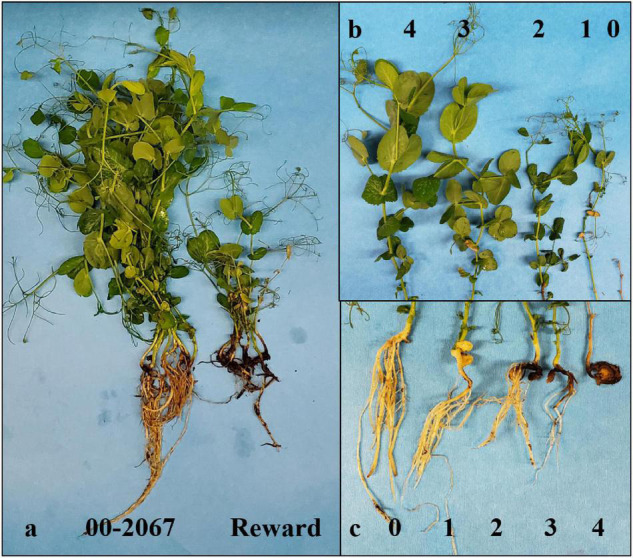
Disease reaction of the parents **(a)** and scales used to rate disease severity **(c)** and vigor **(b).**

Plastic cups (9-cm diameter and 10.5-cm depth) were filled with a sterilized potting mixture (Cell-TechTM, Monsanto, Winnipeg, MB, Canada). In the greenhouse tests with each SSI (S4C, F4A, F037, F039, and FG2), the roots of seven 5-day-old seedlings of the partially resistant parent “00-2067” and the susceptible parent “Reward” were immersed in the conidial suspension for 15 min and transplanted into the soilless mixture in a cup. An aliquot (1 ml) of conidial suspension was pipetted onto the roots before they were covered with the potting mix. The plants were kept in a greenhouse at 20–25°C/15–18°C day/night and a 16-h photoperiod with daily watering to maintain the potting mix at saturation conditions conducive for FRR development. Each experiment was repeated two times. After 3 weeks, disease severity was estimated for the parental cultivars on a scale of 0–4, where: 0 = completely healthy; 1 = brown or black spots on the main root; 2 = lesions covering the main root, but the rootlets still healthy; 3 = lesions spread to the entire root system; and 4 = root totally dead (Figures 1a,c).

### Disease Assessment of Recombinant Inbred Line Population Under Controlled Conditions

The most virulent of the *Fusarium* isolates was used as inoculum to screen the 135 RIL population and parents under greenhouse conditions. The inoculation and the maintenance of the plants were as described above. The pots were arranged in a randomized complete block design (RCBD) with four replicates. The greenhouse experiment was repeated four times. After 3 weeks, plant height (cm) was measured from the base of the stem to the top leaf. Plant vigor was evaluated as a measure of the wilting severity on a scale of 0–4 (4 = plant completely healthy; 3 = thin stem and short height; 2 = brown lesions on stem and yellowing of leaf tips; 1= wilting on stems and leaves; 0 = plant completely dead) (Figure 1b). The plants were then carefully uprooted, washed under standing water, and assessed for disease severity as described above.

### Statistical Analysis of Phenotypic Data

ANOVA was conducted using R software (R Core Team, 2019) for disease severity, vigor, and plant height in four greenhouse environments. The mean and least square mean (LSM) of all traits were calculated for single environments and total data using the package “lsmeans” (Lenth, 2016) in R. To estimate random effects, the best linear unbiased predictors (BLUPs) and heritability were also calculated using the package “Phenotype” (Zhao, 2020) in R. Correlation analysis was conducted within each trait (all variables including means for single environments, LSM, and BLUPs for total data) and among traits (including disease severity, vigor, and plant height) using the package “PerformanceAnalytics” (Peterson et al., 2019) in R, displaying the correlation coefficient, frequency distribution, and dot plot. The *P*-value of the Shapiro–Wilk test was used to determine the normality for each variable using R (R Core Team, 2019).

### Genotyping With Single Nucleotide Polymorphisms and Simple Sequence Repeat Markers

The 13.2K SNP markers and 222 SSR markers, the parents, and the RIL population genotyped by Wu et al. (2021) were used in this study. In brief, SNP genotyping was carried out at TraitGenetics GmbH, Gatersleben, Germany, using an SNP array developed from gene-encoding sequences, which are distributed uniformly across the pea genome (Tayeh et al., 2015). The SSR markers were obtained from Loridon et al. (2005). In the case of the SSR markers, the PCR assays, thermal cycling conditions, and genotyping using an ABI PRISM 3730 x l DNA analyzer (Applied Biosystems, Foster City, CA, United States) were as described by Wu et al. (2021). Filtering of the SNP and SSR was carried out to retain highly polymorphic markers and RIL individuals with > 95% genotyping data, as well as markers that exhibited the expected 1:1 segregation ratio.

### Linkage Map Construction

Linkage analysis was carried out using the filtered SNP and SSR markers, following Wu et al. (2021). This involved the generation of a draft linkage map using the minimum spanning tree map (MSTMap) (Wu et al., 2008) and then refined by MAPMAKER/EXP 3.0 (Lincoln et al., 1992). The Kosambi map function (Kosambi, 1944) was used to calculate the genetic distances (in cM) between the markers. The map construction was carried out with MapChart v. 2.32 (Voorrips, 2002) using the Kosambi map function, of which the linkage groups were assigned to chromosomes based on the consensus SNP map of pea developed by Tayeh et al. (2015). The sequences of the SNP markers flanking the QTLs associated with partial resistance to FRR caused by *F. graminearum* were used in BlastN (*E*-value ≤ E-20) searches of the Pulse Crop Database^1^ to determine their possible functions.

### Quantitative Trait Loci Analysis

Additive-effect QTL analysis was first carried out using the genotypic and phenotypic data (disease severity, vigor, and plant height) from the RILs inoculated with *F. graminearum* (FG2). This was then repeated for the RILs inoculated with *F. avenaceum* (F4A). The analysis was conducted using means for the four single greenhouse experiments, LSM, and BLUPs of the total data by Composite Interval Mapping (CIM) using WinQTL Cartographer v2.5 (Wang et al., 2012). The program was set at 1-cM walking speed; forward and backward regression method; window size, 10 cM; five background cofactors; 1,000 permutations, and *p* < 0.05 (Wang et al., 2012). The LOD score threshold was set at 3 for QTL detection. The confidence interval for each QTL was defined by the consensus region bordered by the four environments.

The QTL names were defined according to the QTL detection studies by Coyne et al. (2015, 2019), where the name of the *Fusarium* isolate was indicated, followed by “*Ps*” = *Pisum sativum*, the first number = the pea linkage group (Tayeh et al., 2015), and the second number = the serial number of the QTL on the linkage group; for example, “*Fg-Ps4.1*” represents the QTL for disease severity caused by *F*. *graminearum* located on linkage group IV of the pea genome. The chromosomes and pseudomolecules were named in accordance with Neumann et al. (2002) and Kreplak et al. (2019), respectively. A similar nomenclature was adopted for vigor (e.g., *Vig-Ps2.1*) and plant height (e.g., *Hgt-Ps2.1*).

Quantitative trait loci identified in at least two of the four environments were classified as stable. The percentage of variation (*R*^2^) was determined for each QTL. Furthermore, QTL with *R*^2^ > 10%, 5–10%, and <5% were arbitrarily classified as major-, moderate-, or minor-effect QTL, respectively. The origins of favorable alleles for individual traits were assigned to different parents, following Wu et al. (2021). Pairwise epistatic interactions were estimated with IciMapping V.4.1 using the ICIM-EPI method (Meng et al., 2015). The significance threshold for major, moderate, and minor was arbitrarily set at *R*^2^ > 15%, 7.5–15%, and <7.5%, respectively. Epistatic-effect QTL were named with the prefix “*E*,” followed by the QTL name and a serial number (e.g., *E.FG-Ps1*, *E.Vig-Ps1*, and *E.Hgt-Ps1*).

## Results

### Preliminary Root Rot Assessment in Parents Against Five *Fusarium* spp.

Between the parental cultivars, “00-2067” developed lower root rot severity than “Reward” in response to each of the five isolates (Supplementary Table 1), confirming that “00-2067” was tolerant, while “Reward” was susceptible. There were significant differences (*p* < 0.001) between the mean root rot values of the tolerant parent “00-2067” and the susceptible parent “Reward”, following inoculation with *F. graminearum* isolate FG2 (Figure 1a) and *F. avenaceum* isolate F4A, while no significant differences were detected following inoculation with the *F. solani*, *F. acuminatum*, and *F. proliferatum* isolates S4C, F037, and F039, respectively (Supplementary Table 1). Therefore, FG2 and F4A were selected to screen the 135 F_8_ RIL population for QTL identification associated with resistance to FRR.

### ANOVA for Disease Severity, Vigor, and Plant Height

The mean root rot severity, vigor, and plant height of the RIL population inoculated with FG2 and F4A are presented in Tables 1, 2. ANOVA indicated that the genotypic effect of disease severity, vigor, and plant height was significant (*p* < 0.001) (Supplementary Tables 2a,b). This suggested that a high proportion of genetic variance was transmitted from parental cultivars to the progenies. Heritability values of 92 and 86% for disease severity and vigor were obtained for plants inoculated with FG2 and F4A, respectively, while heritability values for plant height ranged from 79 to 91% (Supplementary Tables 2a,b). The G × E interactions were significant for disease severity, vigor, and plant height for F4A but not for FG2, while differences among the four greenhouse experiments were significant for both FG2 and F4A (*p* < 0.001) (Supplementary Tables 2a,b).

**TABLE 1:** A statistical summary of phenotypic data for the parental pea cultivars, “00-2067” and “Reward”, and an RIL population inoculated with *Fusarium graminearum* isolate FG2, in four greenhouse experiments, as well as the pooled and the best linear unbiased predictors (BLUPs).

Trait	Abbrev/Experiment	Parental cultivar			RIL population			
		‘00-2067’^a^	‘Reward’^a^	*T*-test (*P*)	RILs^a^	Skewness	Kurtosis	Shapiro-test (*P*)
Root rot severity	DSGH1	1.5 ± 0.7	3.3 ± 0.5	1.1E-02	2.0 ± 0.7	–0.1	–0.7	1.6E-03
Root rot severity	DSGH2	1.3 ± 0.6	3.3 ± 0.5	2.6E-03	1.9 ± 0.7	–0.2	–0.6	1.5E-02
Root rot severity	DSGH3	2.0 ± 1.2	3.5 ± 0.6	3.0E-02	2.2 ± 0.7	0.0	–0.2	6.0E-02
Root rot severity	DSGH4	1.5 ± 0.7	3.0 ± 0.0	1.3E-02	2.1 ± 0.7	0.1	–0.3	3.7E-02
Root rot severity	DSGHPooled	1.6 ± 0.8	3.3 ± 0.5	4.6E-07	2.0 ± 0.6	–0.1	–0.7	6.2E-02
Root rot severity	DSGHBLUPS	1.2	4.1	–	2.0 ± 1.2	–0.1	–0.8	4.6E-02
Vigor	VGH1	4.0 ± 0.0	2.0 ± 0.8	1.5E-02	3.0 ± 0.8	–0.3	–0.7	0.0E + 00
Vigor	VGH2	4.0 ± 0.0	2.5 ± 0.6	3.5E-03	3.0 ± 0.7	–0.3	–0.2	9.5E-06
Vigor	VGH3	3.0 ± 1.2	1.5 ± 0.6	3.0E-02	2.7 ± 0.7	–0.2	–0.2	5.1E-06
Vigor	VGH4	3.5 ± 0.7	2.7 ± 0.6	1.2E-01	2.8 ± 0.8	–0.4	0.6	1.1E-03
Vigor	VGHPooled	3.6 ± 0.8	2.2 ± 0.7	6.0E-05	2.9 ± 0.5	0.0	–0.5	1.1E-01
Vigor	VGHBLUPS	4.2	1.6	–	2.9 ± 1.1	–0.1	–0.4	5.1E-02
Plant height	HGH1	234.5 ± 54.6	177.5 ± 36.1	1.3E-01	217.6 ± 96.3	1.0	0.7	0.0E + 00
Plant height	HGH2	157.3 ± 50.6	120.7 ± 31.5	1.6E-01	231.5 ± 87.4	0.7	0.2	5.6E-05
Plant height	HGH3	155.5 ± 59.5	129.5 ± 26.0	2.3E-01	154.5 ± 84.4	1.0	1.1	8.0E-06
Plant height	HGH4	159.7 ± 6.7	178.5 ± 34.6	2.0R-01	184.6 ± 83.9	0.6	0.5	5.5E-02
Plant height	HGHPooled	176.7 ± 56.6	151.5 ± 36.9	5.1E-02	197.5 ± 68.8	1.1	1.2	0.0E + 00
Plant height	HGHBLUPS	158.9	100.8		197.3 ± 135.5	1.0	0.6	0.0E + 00

*^a^The estimated means of parental cultivars, “Reward” and “00-2067,” as well as RILs, are shown along with plus/minus stand error (SE).*

**TABLE 2:** A statistical summary of phenotypic data for the parental pea cultivars, “00-2067” and “Reward”, and an RIL population inoculated with *Fusarium avenaceum* isolate F4A, in four greenhouse experiments, as well as the pooled and the best linear unbiased predictors (BLUPs) of the greenhouse experiments.

Trait	Abbrev/Experiment	Parental cultivar			RIL population			
		‘00-2067’^a^	‘Reward’^a^	*T*-test (*P*)	RILs^a^	Skewness	Kurtosis	Shapiro-test (*P*)
Root rot severity	DSGH1	1.0 ± 0.0	3.3 ± 0.5	5.30E-05	2.2 ± 0.9	0.2	–0.9	7.77E-05
Root rot severity	DSGH2	1.0 ± 0.8	3.3 ± 0.5	1.66E-03	2.3 ± 0.9	0.3	–0.6	1.35E-04
Root rot severity	DSGH3	1.3 ± 0.5	3.5 ± 0.6	5.30E-04	2.5 ± 0.9	0.0	–1.0	2.86E-06
Root rot severity	DSGH4	1.0 ± 0	3.0 ± 0.0	1.36E-03	2.4 ± 0.9	0.0	–0.7	1.38E-02
Root rot severity	DSGHPooled	1.1 ± 0.4	3.3 ± 0.4	2.63E-13	2.4 ± 0.7	0.0	–0.6	1.20E-01
Root rot severity	DSGHBLUPS	1.0	3.0		2.3 ± 0.9	0.1	–1.0	8.07E-05
Vigor	VGH1	4.0 ± 0.0	1.7 ± 0.5	5.26E-05	2.6 ± 1.1	–0.5	–0.6	5.96E-08
Vigor	VGH2	3.7 ± 0.5	2.0 ± 1.4	2.92E-02	2.6 ± 1.1	–0.9	–0.1	0.00E + 00
Vigor	VGH3	3.5 ± 0.6	1.2 ± 1.5	1.56E-02	2.4 ± 1.2	–0.5	–0.7	0.00E + 00
Vigor	VGH4	4.0 ± 0.0	2.5 ± 0.6	1.01E-03	2.5 ± 1.1	–0.4	–1.0	1.79E-07
Vigor	VGHPooled	3.8 ± 0.5	1.9 ± 1.1	1.06E-07	2.5 ± 0.9	–0.5	–0.6	9.89E-06
Vigor	VGHBLUPS	4.0	1.9		2.6 ± 1	–0.6	–0.3	5.96E-08
Plant height	HGH1	210.8 ± 128.2	118.3 ± 100.2	1.49E-01	196.8 ± 87.9	0.7	0.6	1.35E-03
Plant height	HGH2	174.5 ± 104.8	193.5 ± 104.5	4.03E-01	194.5 ± 88.5	0.6	1.3	9.74E-02
Plant height	HGH3	159.5 ± 13.5	125.0 ± 98.9	2.58E-01	171.8 ± 80	0.4	0.3	3.76E-01
Plant height	HGH4	210.0 ± 53.1	194.0 ± 38.4	3.00E-01	180.8 ± 80.1	0.7	0.7	8.17E-03
Plant height	HGHPooled	188.7 ± 82.8	157.7 ± 88.5	1.54E-01	185.2 ± 64	0.6	0.5	9.79E-02
Plant height	HGHBLUPS	208.0	133.1		186.6 ± 122.6	0.6	1.0	5.72E-02

*^a^The estimated means of parental cultivars, “Reward” and “00-2067”, as well as RILs, are shown along with plus/minus stand error (SE).*

### Root Rot, Vigor, and Plant Height of Parents and the Recombinant Inbred Line Population Inoculated With FG2

**FIGURE 2:**
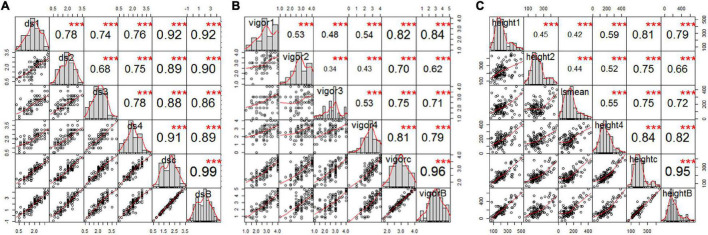
Correlation analysis of estimated mean of four single greenhouse experiments, BLUPs, and combined total data for **(A)** root rot severity, **(B)** vigor, and **(C)** height of pea inoculated with FG2, illustrating the significant correlation among all variables for each trait. The bar graphs indicate the frequency distributions across the diagonal. The correlation coefficients with a significance level (* indicates *p* < 0.05; ** indicates *p* < 0.01; *** indicates *p* < 0.001) and scatter plots between pairs are shown above and below the diagonal, respectively.

Estimated disease severity values (±SE) on the parental cultivar “00-2067” inoculated with FG2 were 1.5 ± 0.7, 1.3 ± 0.6, 2 ± 1.2 and 1.5 ± 0.7 for the four greenhouse experiments, 1.6 ± 0.8 for LSM and 1.2 for the BLUPs. This was comparable with the estimated mean of 1.1 ± 0.4 obtained in the preliminary screening of the parents (Table 1 and Supplementary Table 1). On the other hand, the estimated disease severity values (±SE) for “Reward” were 3.3 ± 0.5, 3.3 ± 0.5, 3.5 ± 0.6, and 3.0 ± 0.0 for the four greenhouse experiments, 3.3 ± 0.5 for LSM and 4.1 for the BLUPs; these values were also comparable to the estimated mean of 3.3 ± 0.4 obtained in the preliminary screening (Table 1 and Supplementary Table 1). A *t*-test indicated a significant difference between the parents for disease severity in all four experiments. Frequency distribution (Figure 2A) indicated that the disease severity data of the RILs in the four experiments were continuous, but only DSGH3 and DSGHC followed a normal distribution based on the Shapiro–Wilk test (Table 1). High correlation coefficients, ranging from 68 to 99%, were found for disease severity among the single experiments, pooled, and BLUPs (Figure 2A). The differences in vigor between the parents inoculated with FG2 were significant, except for VGH4. The parental cultivar, “00-2067” had estimated means (±SE) of 4.0 ± 0.0, 4.0 ± 0.0, 3.0 ± 1.2 and 3.5 ± 0.7 for the four greenhouse experiments and 3.6 ± 0.8 for the pooled data. In the case of “Reward”, the estimated means (±SE) were 2.0 ± 0.8, 2.5 ± 0.6, 1.5 ± 0.6 and 2.7 ± 0.6 for the four greenhouse experiments and 2.2 ± 0.7 for the pooled data. The BLUPs for the parental cultivars “00-2067” and “Reward” were 4.2 and 1.6, respectively (Table 1). The Shapiro–Wilk test indicated that the RIL population vigor data for the four greenhouse experiments did not follow a normal distribution, except for VGHC (Figure 2B). A significant correlation (0.34 < *r* < 0.96, *p* < 0.001) existed among the single experiments, pooled, and BLUPs for vigor (Figure 2B). The height of “00-2067” plants inoculated with FG2 was greater than plants of “Reward” for the means in the single environments, LSM, and BLUPs, although the differences were not significant based on a *t*-test. The estimated means in single conditions, LSM, and BLUP for plant height (±SE) of “00-2067” were 234.5 ± 54.6 cm, 157.3 ± 50.6 cm, 155.5 ± 59.5 cm, 159.7 ± 6.7 cm, 176.7 ± 56.6 cm and 158.9 cm, respectively. For “Reward”, the plant heights were 177.5 ± 36.1 cm, 120.7 ± 31.5 cm, 129.5 ± 26. cm, 178.5 ± 34.6 cm, 151.5 ± 36.9 cm, and 100.8 cm, respectively. The frequency distribution of plant height of the RIL population for all six variables was not normal and slightly skewed (Table 1 and Figure 2C). A high correlation (0.42 < *r* < 0.95, *p* < 0.001) was found for plant height among the single experiments, pooled, and BLUPs data (Figure 2C).

Collectively, the correlation analysis among traits indicated that root rot caused by FG2 was negatively correlated with vigor and plant height. High correlation coefficients were detected between disease severity and vigor in all conditions (–0.65 < *r* < –0.90, *p* < 0.001), indicating the adverse effect of FG2 on root and aboveground growth. Plant height showed low to moderate correlation with disease severity (–0.22 < *r* < –0.35, *p* < 0.05) and vigor (0.19 < *r* < 0.38, *p* < 0.05).

### Root Rot, Vigor, and Plant Height of Parents and the RIL Population Inoculated With F4A

**FIGURE 3:**
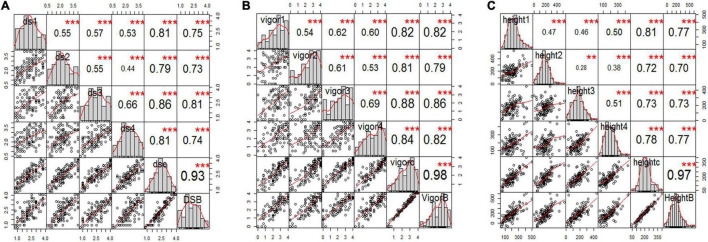
Correlation analysis among three pea root rot disease-related traits of **(A)** root rot severity, **(B)** vigor, and **(C)** plant height for all six variables, including the estimated mean of four single greenhouse studies, combined total data, and BLUPs (e.g., panels ds1, ds2, ds3, ds4, dsc, and dsB for disease severity) inoculated with F4A. The bar graphs indicate the frequency distributions across the diagonal. The correlation coefficients with a significance level (* indicates *p* < 0.05; ** indicates *p* < 0.01; *** indicates *p* < 0.001) and scatter plots between pairs are shown above and below the diagonal, respectively.

The estimated means (±SE) of disease severity for “00-2067” were 1.0 ± 0.0, 1.0 ± 0.8, 1.3 ± 0.5, 1.0 ± 0.0, 1.1 ± 0.4, and 1.0, while, for “Reward”, they were 3.3 ± 0.5, 3.3 ± 0.5, 3.5 ± 0.6, 3.0 ± 0.0, 3.3 ± 0.4, and 3.0 for DSGH1, DSGH2, DSGH3, DSGH4, LSM of pooled data, and BLUPs, respectively (Table 2). These values were comparable to the estimated means (±SE) of 1.8 ± 0.5 and 2.8 ± 0.2 for disease severity obtained in the preliminary screening of the parents (Table 2 and Supplementary Table 1). *t*-tests indicated significant differences between estimated means of the parental cultivars “00-2067” and “Reward” inoculated with F4A. The Shapiro–Wilk test indicated that only the root rot data of the RIL population for DSGH4 and DSGH Pooled followed a normal distribution (Table 2), although the data for the four greenhouse experiments were continuous (Figure 3A). The correlation coefficient between the experiments ranged from 0.44 to 0.93 (*p* < 0.001) (Figure 3A). Based on the *t*-tests, the parental cultivar “00-2067” inoculated with F4A had significantly greater vigor than “Reward.” The estimated vigor values (±SE) for “00-2067” were 4.0 ± 0.0, 3.7 ± 0.5, 3.5 ± 0.6, and 4 ± 0 for the four individual greenhouse experiments, 3.8 ± 0.5 for LSM for the pooled data and 4.0 for BULPs of the pooled greenhouse experiments (Table 2). The estimated vigor values (±SE) for “Reward” were 1.7 ± 0.5, 2.0 ± 1.4, 1.2 ± 1.5, and 2.5 ± 0.6 for the individual greenhouse experiments, 1.9 ± 1.1 for LSM, and 1.9 for the BULPs of the pooled greenhouse experiments. All vigor variables for the RIL population were continuous with slight left skewness (–0.4∼–0.9) (Figure 3B). Additionally, the data did not follow a normal distribution based on the Shapiro–Wilk test (Table 2). The correlation coefficient between the experiments ranged from 0.54 to 0.98 (*p* < 0.001) (Figure 3B).

In contrast to vigor, the difference in plant height of the parental cultivars inoculated with F4A was not significant based on the *t*-test. The estimated plant height for “00-2067” for the individual experiments, LSM, and BLUP was 210.8 ± 128.2 cm, 174.5 ± 104.8 cm, 159.5 ± 13.5 cm, 210.0 ± 53.1 cm, 188.7 ± 82.8 cm, and 208.0 cm, respectively. The estimated plant height for “Reward” was 118.3 ± 100.2 cm, 193.5 ± 104.5 cm, 125. ± 98.9 cm, and 194.0 ± 38.4 cm for the individual experiments, 157.7 ± 88.5 cm for LSM and 133.1 cm for the BLUP. The frequency distribution for the RIL population was continuous and slightly skewed to the right. In addition, HGH2, HGH3, HGH Pooled, and HGH BLUPs followed a normal distribution (Table 2 and Figure 3C). Plant height variables were also significantly correlated (0.28 < *r* < 0.97, *p* < 0.01) (Figure 3C).

The correlation among the traits for plants inoculated with F4A was similar to that of plants inoculated with FG2. Disease severity was highly correlated with vigor (–0.88 < *r* < –0.95, *p* < 0.001) and with plant height (–0.48 < *r* < –0.63, *p* < 0.001). Plant height was positively correlated with vigor (0.57 < *r* < 0.62, *p* < 0.001).

### Genetic Map Construction and Quantitative Trait Loci Analysis

Linkage grouping, the distribution of markers, map length, and marker density of 2999 (2978 SNP + 21 SSR) retained markers were as described by Wu et al. (2021). The marker distribution in this study was compared with the seven chromosomes of pea as determined by Neumann et al. (2002), linkage groups as determined by Tayeh et al. (2015), and pseudomolecules of pea (Kreplak et al., 2019). The genetic map spanned 1704.1 cM and contained an average marker density of 1.8 markers/cM (Wu et al., 2021). The QTL analysis was conducted with 1,422 unique markers, which represented 10.5% of the markers used for genotyping (Wu et al., 2021).

### Additive-Effect Quantitative Trait Loci Analysis

No significant QTL (LOD > 3.0) for disease severity, vigor, and plant height were detected for the RILs inoculated with *F. avenaceum* isolate F4A. As such, no QTL likelihood profiles are shown. In the case of RILs inoculated with *F. graminearum* isolate FG2, a total of 11 QTL were detected for the three parameters and six variables (i.e., GH1, GH2, GH3, GH4, LSM, and BLUPs) by the CIM using Win QTL Cartographer v2.5 (Wang et al., 2012; Table 3). Five of the 11 QTL were identified for disease severity, whereas three QTL each were detected for vigor and plant height. The QTL had LOD scores ranging from 3.0 to 14.4 and the percentage of phenotypic variation (*R*^2^) values ranging from 4.05 to 36.35% (Table 3). Based on the *R*^2^ values, two, six, and three of the QTL were considered major, moderate, or minor effect, respectively. Six of the 11 QTL were identified in two or more environments and hence could be considered stable, while the remaining five QTL were detected in single experiments and hence could be considered unstable.

**TABLE 3:** A summary of the QTL associated with Fusarium root rot severity, vigor, and plant height in 128 F_8_-derived recombinant inbred pea lines from the cross between the cultivars “Reward” × “00-2067” under greenhouse (GH) conditions.

Identified QTL	Trait	Environment	LG Analysis (Present study)	Chrom^α^/LG^β^	Peak (cM)	Confidence interval(cM)	Left Marker	Right marker	LOD	Additive	*R*^2^(%)
*Fg-Ps3.1*	Root rot severity	GH Expt 1	4	Chrom5/LGIII	311.9	307.9–316.5	AA5	PsCam036163_21311_1095	3.9	–0.2409	9.88
*Fg-Ps3.2*	Root rot severity	GH Expt 4	4	Chrom5/LGIII	338.2	334.9–341.4	PsCam036163_21311_1095	PsCam042783_26826_1395	3.5	–0.2153	9.62
*Fg-Ps4.1*	Root rot severity	GH Expt 1	5	Chrom4/LGIV	71.7	63.7–74.4	PsCam050913_33466_1250	PsCam048871_31524_450	3.0	–0.2121	9.10
	Root rot severity	GH Expt 2	5	Chrom4/LGIV	61.3	59.3–69.2	PsCam001381_1152_437	PsCam042375_26443_3427	3.8	–0.2229	10.57
*Fg-Ps4.2*	Root rot severity	GH Expt 3	5	Chrom4/LGIV	80.2	74.0–85.2	AA239	PsCam057281_37909_2940	5.9	–0.2344	11.26
	Root rot severity	GH Expt 4	5	Chrom4/LGIV	80.2	75.4–85.2	AA239	PsCam057281_37909_2940	4.1	–0.2526	13.17
	Root rot severity	Pooled	5	Chrom4/LGIV	79.2	75.4–85.2	AA239	PsCam057281_37909_2940	5.1	–0.2492	15.44
	Root rot severity	BLUPs	5	Chrom4/LGIV	79.2	75.4-85.2	AA239	PsCam057281_37909_2940	3.7	–0.3838	10.02
*Fg-Ps5.1*	Root rot severity	GH Expt 1	7	Chrom3/LGV	5.2	0.9–9.2	PsCam059449_39630_321	PsCam011153_7569_125	5.5	0.3036	14.22
*Vig-Ps3.1*	Vigor	GH Expt 4	4	Chrom5/LGIII	68.9	67.1–70.5	PsCam013763_9362_423	AD270	4.9	–0.1423	4.05
*Vig-Ps3.2*	Vigor	GH Expt 2	4	Chrom5/LGIII	312.0	307.8–316.8	AA5	PsCam036163_21311_1095	3.0	0.1910	9.53
	Vigor	GH Expt 3	4	Chrom5/LGIII	316.1	310.4–320.4	AA5	PsCam036163_21311_1095	3.3	0.2582	11.22
	Vigor	Pooled	4	Chrom5/LGIII	312.6	310.4–316.5	AA5	PsCam036163_21311_1095	4.6	0.1938	12.13
	Vigor	BLUPs	4	Chrom5/LGIII	312.6	307.5–316.5	AA5	PsCam036163_21311_1095	4.2	0.3736	11.92
*Vig-Ps4.1*	Vigor	GH Expt 1	5	Chrom4/LGIV	68.0	63.5–69.9	PsCam050913_33466_1250	PsCam042375_26443_3427	4.4	0.2728	13.50
	Vigor	GH Expt 2	5	Chrom4/LGIV	60.3	58.0–70.7	PsCam000712_620_237	PsCam042375_26443_3427	3.2	0.2060	10.42
	Vigor	GH Expt 4	5	Chrom4/LGIV	71.5	70.5–73.2	PsCam042375_26443_3427	AA239	4.5	0.2437	9.19
	Vigor	Pooled	5	Chrom4/LGIV	61.3	58.8–63.5	PsCam000712_620_237	PsCam057555_38139_296	4.4	0.1868	11.59
	Vigor	BLUPs	5	Chrom4/LGIV	61.3	59.3–63.5	PsCam001381_1152_437	PsCam057555_38139_296	3.8	0.3474	10.51
*Hgt-Ps3.1*	Height	GH Expt 1	4	Chrom5/LGIII	288.6	288.3–291.7	PsCam020937_11699_2576	AA5	14.4	–62.31	36.35
	Height	GH Expt 2	4	Chrom5/LGIII	287.6	286.8–293.7	PsCam020937_11699_2576	AA5	4.7	–33.90	12.90
	Height	GH Expt 4	4	Chrom5/LGIII	287.6	286.8–295.2	PsCam020937_11699_2576	AA5	3.3	–27.27	9.94
	Height	Pooled	4	Chrom5/LGIII	287.6	286.8–292.4	PsCam020937_11699_2576	AA5	6.2	–33.24	20.96
	Height	BLUPs	4	Chrom5/LGIII	287.6	286.8–291.4	PsCam020937_11699_2576	AA5	9.4	–71.63	23.97
*Hgt-Ps7.1*	Height	GH Expt 1	9	Chrom7/LGVII	92.2	85.3–102.1	PsCam039854_24711_656	PsCam046792_30096_853	10.1	46.60	20.04
	Height	Pooled	9	Chrom7/LGVII	92.2	84.5–115.3	PsCam056683_37453_248	PsCam021891_12310_347	4.9	28.68	13.54
	Height	BLUPs	9	Chrom7/LGVII	92.2	81.2–102.5	PsCam035831_20992_561	PsCam042171_26273_1937	4.5	52.40	7.04
*Hgt-Ps7.2*	Height	GH Expt 2	9	Chrom7/LGVII	154.3	143.8–167.5	PsCam002756_2184_427	PsCam045262_28962_162	5.1	34.63	13.63
	Height	GH Expt 4	9	Chrom7/LGVII	144.3	142.3–151.9	PsCam002756_2184_427	PsCam011213_7616_1104	4.4	32.77	13.85
	Height	Pooled	9	Chrom7/LGVII	157.7	148.8–168.0	AB91	PsCam045262_28962_162	4.4	27.87	14.06

*^α^Pea chromosomes named according to Neumann et al. (2002) and ^β^Pea linkage groups named according to Tayeh et al. (2015).*

**FIGURE 4:**
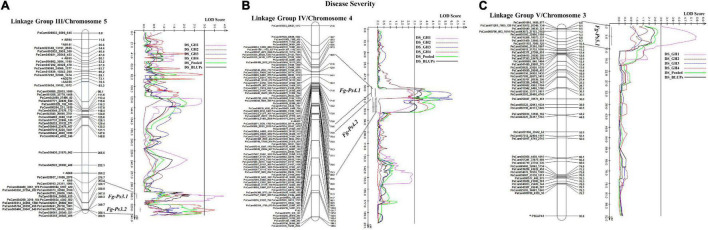
Identified QTL and the linkage map of pea LG III (Chrom 5), IV (Chrom 4), and V (Chrom 3) associated with partial resistance to *Fusarium graminearum* in an F_8_ RIL derived from “Reward” × “002067.” The LOD scores are indicated on the *x*-axis, while the genetic distances (in cM) are indicated on the *y*-axis. **(A)** Two minor-effect QTL, *Fg-Ps3.1* and *Fg-Ps3.2*, on LG III (Chrom5) were detected in greenhouse Experiments 1 and 4, respectively. **(B)** Two stable, moderate-effect QTL, *Fg-Ps4.1* and *Fg-Ps4.2*, were located on LG IV (Chrom4) and identified in greenhouse Experiments 1 and 2 and 3 and 4, respectively. **(C)** Another moderate-effect QTL, *Fg-Ps5.1*, on LG V (Chrom5) was detected only in greenhouse Experiment 1.

The most stable QTL for partial resistance to *F. graminearum* isolate FG2, *Fg-Ps4.1*, and *Fg-Ps4.2* were located in the middle of Chrom4/LGIV at positions 59.3–74.4 cM and 74.0–85.2 cM, respectively (Figure 4B). The 15.1-cM and 11.2-cM genomic regions delimiting these two QTL were flanked by the SNP markers PsCam048871_31524_450 and PsCam001381_1152_437 and the SSR marker AA239 and SNP marker PsCam057281_37909_2940, respectively (Figure 4B). Both *Fg-Ps4.1* and *Fg-Ps4.2* exhibited a moderate effect, with the percentage variance ranging from 9.1 to 15.4% (Table 3). Two other moderate-effect but unstable QTLs, *Fg-Ps3.1* (located on the bottom segment (307.9–316.5 cM) of Chrom5/LGIII and with flanking markers of AA5 and PsCam036163_21311_1095) and *Fg-Ps3.2* (located distal to *Fg-Ps3.1* and with flanking markers PsCam036163_21311_1095 and PsCam042783_26826_1395) explained 9.62–9.88% of the total variance (Figure 4A). Another unstable QTL, *Fg-Ps5.1* [detected on the top part (0.9–9.2 cM) of Chrom3/LGV and flanked by the SNP markers PsCam059449_39630_321 and PsCam011153_7569_125] explained 14.2% of the total variance in greenhouse Experiment 1 (Figure 4C). Four of the QTL for disease severity (with the exception of *Fg-Ps5.1*) had a negative additive effect, indicating that genomic regions for resistance in *Fg-Ps4.1, Fg-Ps4.2, Fg-Ps3.1*, and *Fg-Ps3.2* originated from “00-2067,” while *Fg-ps5.1* derived its resistance from “Reward” (Table 3).

**FIGURE 5:**
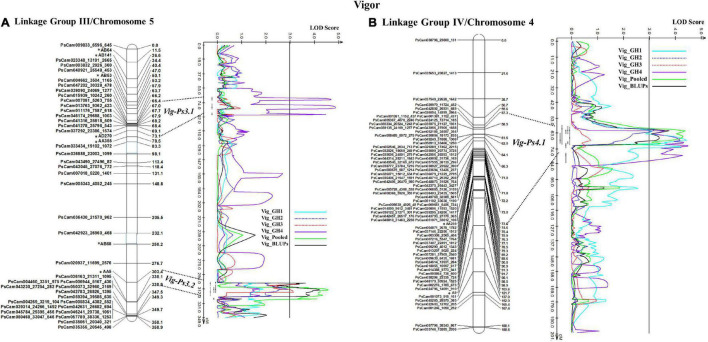
The QTL likelihood profile and the linkage map of pea LG III (Chrom5) and IV (Chrom 4) for vigor in an F_8_ RIL of the cross “Reward” × “002067.” The LOD scores are indicated on the *x*-axis, while the genetic distances (in cM) are indicated on the *y*-axis. **(A)** Minor-effect QTL *Vig-Ps3.1* on LG III (Chrom5) was detected for vigor only in greenhouse Experiment 4. Another QTL *Vig-Ps3.2* was identified multiple times in greenhouse Experiments 2 and 3, as well as in the BLUPs and pooled data. **(B)** One minor-moderate-effect QTL, *Vig-Ps4.1*, was identified on LG IV (Chrom 4) greenhouse Experiments 1, 2, and 5, as well as the pooled data and BLUPs.

The stability of the QTL for vigor was in the order *Vig-Ps4.1* on Chrom4/LGIV (GH1, GH2, and GH4, *R*^2^ = 9.19 to 13.5%) > *Vig-Ps3.2* (GH2 and GH3, *R*^2^ = 9.53% to 12.13%) > *Vig-Ps3.1* (GH4, *R*^2^ = 4.05%) both on Chrom5/LGIII (Table 3). The QTL *Vig-Ps4.1* was located on Chrom4/LGIV from 58.0 cM to 73.2 cm between the SNP marker PsCam000712_620_237 and the SSR marker AA239 (Figure 5B). *Vig-Ps3.2*, which was located 307.8-316.5 cM on the bottom of Chrom5/LGIII, was flanked by the SSR marker AA5 and the SNP marker PsCam036163_21311_1095; *Vig-Ps3.1*, which was located on the top segment (67.1–70.5 cM) of the same chromosome or linkage group, was flanked by the SNP marker PsCam013763_9362_423 and the SSR marker AD270 (Figure 5). The two stable QTL, *Vig-Ps4.1* and *Vig-Ps3.2*, had a positive additive effect, indicating that the alleles for vigor originated from “00-2067.” In contrast, *Vig-Ps3.1* has a negative additive effect, indicating that the alleles originated from “Reward” (Table 3).

**FIGURE 6:**
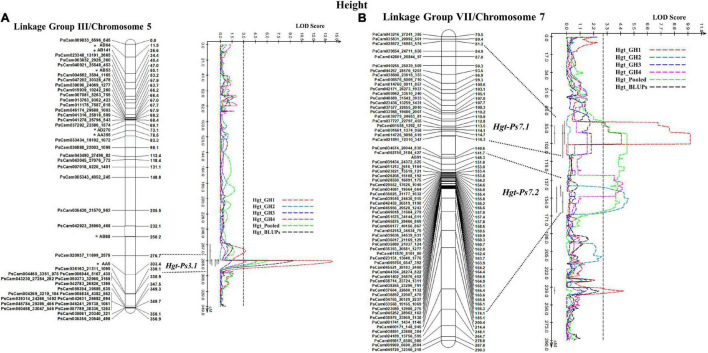
The QTL likelihood profile and the linkage map of Peas III (Chrom5) and VII (Chrom7) for plant height in an F_8_ RIL of the cross “Reward” × “002067.” The LOD scores are indicated on the *x*-axis, while the genetic distances (in cM) are indicated on the *y*-axis. **(A)** One stable QTL, *Hgt-Ps3.1* on LGIII (Chrom5), was detected by all variables except greenhouse Experiment 3, with a minor to major effect. **(B)** Two QTL were detected on LGVII (Chrom7); the minor-major-effect QTL *Hgt-Ps7.1* was detected in greenhouse Experiment 1, as well as in the pooled data and BLUPs, while the moderate-effect QTL *Hgt-Ps7.2* was detected in greenhouse Experiments 2 and 4 and the pooled data.

In the case of plant height, the most stable QTL, *Hgt-Ps3.1*, was detected in three of the four experiments (GH1, GH2, and GH4; *R*^2^ = 9.94–36.35%). This QTL was located on the bottom segment of Chrom5/LGIII (Figure 6) and was flanked by the SNP marker PsCam020937_11699_2576 and the SSR marker AA5 (Figure 6A). The second most stable QTL, *Hgt-Ps7.2*, was detected across two (GH2 and GH4) of four greenhouse experiments (*R*^2^ = 7.04–20.04%). These QTL were located 142.3–168.0 cM on Chrom7/LGVII and were flanked by the SNP markers PsCam002756_2184_427 and PsCam045262_28962_162 (Table 3 and Figure 6B). *Hgt-Ps7.1*, which was flanked by the SNP markers PsCam035831_20992_561 and PsCam021891_12310_347 (81.2–115.3 cM) (Figure 6B), was detected in only one environment (GH1) on the same chromosome (*R*^2^ = 13.63–14.06). The additive effect was negative for *Hgt-Ps3.1*, but positive for *Hgt-Ps7.1* and *Hgt-Ps7.2* (Table 3). This suggested that the QTL for height on Chrom5/LGIII was derived from “Reward”, while the QTL on Chrom7/LGVII originated from “00-2067.”

### Epistatic Quantitative Trait Loci Analyses

Two hundred eight putative digenic epistatic pairs were identified using all variables for disease severity, vigor, and plant height. These comprised 65 (12-24) for disease severity, 57 (10–21) for vigor, and 86 (15–28) for plant height. The 208 putative digenic interactions consisted of one major epistatic effect (PVE ≥ 15%), 13 moderate epistatic effects (7.5% ≤ PVE ≤ 15%), and 194 minor epistatic effects (PVE ≤ 7.5%). BLUPs for disease severity, vigor, and plant height detected 20, 18, and 21 putative digenic interactions, respectively. Within the 59 digenic interactions among BLUPs of all traits, epistatic analysis identified one major QTL pair, three moderate QTL pairs, and 55 minor QTL pairs (Table 4). In contrast, LSM of the pooled data detected 23 digenic interactions for disease severity, 14 for vigor, and 19 for plant height. The total 56 pairs included seven moderate epistatic-effect QTLs and 49 minor-effect QTLs.

**TABLE 4:** A summary of the major and moderate digenic epistatic interactions (QTL × QTL) detected for Fusarium root rot severity, vigor, and plant height in four greenhouse experiments with pea.

Identified epistatic-effect QTL	Trait	Environment*	LG	QTL 1 position	Left Marker QTL 1	Right marker QTL 1	LG	QTL 2 position	Left Marker QTL 2	Right marker QTL 2	*R*^2^ (%)	Linked additive-effect QTL
*E.FG2-Ps1*	Fusarium root rot	GH Expt 1	III	130	PsCam055659 _36655_1042	PsCam038445_23479 _407	III	315	AA5	PsCam036163_213 11_1095	9.5	*Fg-Ps3.1*
*E.FG2-Ps2*	Fusarium root rot	GH Expt 1	III	345	PsCam004460_ 3351_975	PsCam042783_2682 6_1395	IV	175	PsCam001246_105 0_252	PsCam005487_415 1_529	7.8	*Fg-Ps3.2*
*E.FG2-Ps3*	Fusarium root rot	GH Expt 2	II	240	PsCam052149_ 34531_162	PsCam005624_424 5_1053	III	175	PsCam005343_405 2_245	PsCam035416_2060 3_89	12.4	-
*E.FG2-Ps4*	Fusarium root rot	GH Expt 2	III	135	PsCam007018_ 5220_1401	PsCam006445_480 0_1399	III	220	PsCam035416_206 03_89	PsCam042923_269 60_468	10.1	–
*E.FG2-Ps5*	Fusarium root rot	GH Expt 3	III	160	PsCam005343_ 4052_245	PsCam035416_2060 3_89	III	220	PsCam035416_206 03_89	PsCam042923_269 60_468	9.0	–
*E.FG2-Ps6*	Fusarium root rot	Pooled GH Expts	III	170	PsCam005343_ 4052_245	PsCam035416_2060 3_89	III	215	PsCam035416_206 03_89	PsCam042923_ 26960_468	9.2	–
*E.FG2-Ps7*	Fusarium root rot	BLUPs GH Expts	III	170	PsCam005343_ 4052_245	PsCam035416_2060 3_89	III	215	PsCam035416_206 03_89	PsCam042923_ 26960_468	10.5	–
*E.FG2-Ps8*	Fusarium root rot	BLUPS GH Expts	III	245	PsCam042923_ 26960_468	AB68	III	90	PsCam005789_43 53_36	PSGAPA1	7.5	–
*E.Vig-Ps1*	Vigor	GH Expt 1	III	175	PsCam005343_ 4052_245	PsCam035416_206 03_89	III	375	PsCam019069_113 10_393	PsCam048824_3147 7_2784	11.2	–
*E.Vig-Ps2*	Vigor	GH Expt 2	I	55	PsCam037897_ 22954_2120	PsCam057553_38 137_135	III	170	PsCam005343_ 4052_245	PsCam035416_2060 3_89	10.0	–
*E.Vig-Ps3*	Vigor	GH Expt 2	III	170	PsCam005343_ 4052_245	PsCam035416_206 03_89	III	215	PsCam035416_206 03_89	PsCam042923_269 60_468	12.1	–
*E.Vig-Ps4*	Vigor	GH Expt 4	I	55	PsCam037897_ 22954_2120	PsCam057553_381 37_135	III	190	PsCam005343_405 2_245	PsCam035416_206 03_89	8.5	–
*E.Vig-Ps5*	Vigor	GH Expt 4	III	195	PsCam005343_ 4052_245	PsCam035416_20 603_89	III	215	PsCam035416_206 03_89	PsCam042923_269 60_468	8.7	–
*E.Vig-Ps6*	Vigor	GH Expt 4	III	185	PsCam005343_ 4052_245	PsCam035416_2060 3_89	VII	50	PsCam017623_1085 8_46	PsCam000168_14 5_1509	9.6	–
*E.Vig-Ps7*	Vigor	Pooled GH Expt	III	220	PsCam035416_ 20603_89	PsCam042923_269 60_468	III	315	AA5	PsCam036163_2131 1_1095	12.6	*Fg-Ps3.1*
*E.Vig-Ps8*	Vigor	Pooled GH Expt	III	390	PsCam029411_ 17551_1348	PsCam042665_267 15_153	V	85	PsCam005789_435 3_36	PSGAPA1	7.8	–
*E.Vig-Ps9*	Vigor	Pooled GH Expt	III	215	PsCam035416_ 20603_89	PsCam042923_269 60_468	VI	85	PsCam042529_2658 4_303	PsCam037575_226 53_1339	10.0	–
*E.Vig-Ps10*	Vigor	BLUPs GH Expt	III	85	PsCam050501_ 33079_1023	PsCam036791_219 14_640	III	170	PsCam005343_405 2_245	PsCam035416_206 03_89	9.8	–
*E.Hgt-Ps1*	Plant height	GH Expt 1	III	340	PsCam004460_ 3351_975	PsCam042783_268 26_1395	III	345	PsCam004460_ 3351_975	PsCam042783_2682 6_1395	31.2	*Fg-Ps3.2*
*E.Hgt-Ps2*	Plant height	GH Expt 1	III	340	PsCam004460_ 3351_975	PsCam042783_268 26_1395	VII	60	PsCam001066_9 09_911	PsCam056652_3743 0_307	9.9	*Fg-Ps3.2*
*E.Hgt-Ps3*	Plant height	GH Expt 2	III	70	PsCam004460_ 3351_975	PsCam042783_2682 6_1395	III	345	PsCam004460_3 351_975	PsCam042783_2682 6_1395	13.2	*Vig-Ps3.1*and*Fg-Ps3.2*
*E.Hgt-Ps4*	Plant height	Pooled GH Expt	III	340	PsCam042923_ 26960_468	AB68	III	335	PsCam004460_ 3351_975	PsCam042783_268 26_1395	13.5	*Fg-Ps3.2*
*E.Hgt-Ps5*	Plant height	Pooled GH Expt	III	340	PsCam004460_ 3351_975	PsCam042783_268 26_1395	IV	25	PsCam054029_35 722_104	PsCam037549_226 28_1642	8.9	*Fg-Ps3.2*
*E.Hgt-Ps6*	Plant height	Pooled GH Expt	III	340	PsCam004460_ 3351_975	PsCam042783_268 26_1395	VII	60	PsCam001066_9 09_911	PsCam056652_37 430_307	8.9	*Fg-Ps3.2*
*E.Hgt-Ps7*	Plant height	BLUPs GH Expt	III	340	PsCam004460_ 3351_975	PsCam042783_268 26_1395	III	345	PsCam004460_33 51_975	PsCam042783_268 26_1395	19.1	*Fg-Ps3.2*

*GH, greenhouse; Expt, experiment.

Twenty-five digenic epistatic interactions with major and moderate effects were identified by 33 flanking markers, of which 10 epistatic-effect QTL with 14 flanking markers were linked to three additive-effect QTL (*Fg-Ps3.1, Fg-Ps3.2*, and *Vig-Ps3.1*). The remaining 15 epistatic QTL were not related to any of the additive-effect QTL (Table 4). Eight of the 10 epistatic-effect QTL were linked to *Fg-Ps3.2*, including the most significant QTL pairs, *E.Hgt-Ps1* (*R*^2^ = 31.2%), followed by *E.Hgt-Ps7* (*R*^2^ = 19.1%) and *E.Hgt-Ps4* (*R*^2^ = 13.5%). The fourth was *E.Hgt-Ps3* (*R*^2^ = 13.5%), which was linked to *Fg-Ps3.2* and *Vig-Ps3.1*. Only *E.Fg-Ps7* and *E.Vig-Ps7* were linked to Fg-Ps3.1, showing moderate epistatic effect (*R*^2^ = 9.5% and *R*^2^ = 12.6%, respectively).

### Candidate Genes

The QTL associated with partial resistance to *F. graminearum* on Chrom5/LGIII and Chrom4/LGVI flanked four and 74 candidate genes, respectively (Supplementary Table 3). Fifteen of the 74 genes were related to plant defense mechanisms. These included UDP formation and transportation, the integral component of membrane proteins, histone-lysine *N*-methyltransferase, phospholipid transport, actin cytoskeleton, calcium-ion binding, methyltransferase, UBQ-conjugating enzyme/RWD, AP-4 adaptor complex, oxidoreductase, acyl group transferases, hydrolases, G protein-coupled receptors, and protein involved in phosphorylation and proteolysis. Some of the genes were involved in pathways related to plant defense mechanisms. Psat4g125440 is involved in cellulose biosynthesis, while Psat4g111280, Psat4g110800, Psat4g108480, and Psat4g102720 are involved in protein ubiquitination.

## Discussion

Commercial farming in Canada is characterized by short rotations of cereal crops with canola and, to a limited extent, pulse crops. Disease surveys in Canada have identified *Fusarium* species as the most frequently isolated fungi from all crops surveyed for root rot severity (Chang, unpublished data). *Fusarium poae* was predominant in FHB-infected kernels, followed by *F. graminearum*; other *Fusarium* species were less common in infected kernels (Banik et al., 2019; Xue et al., 2019; Ziesman et al., 2019). The predominant *Fusarium* spp. isolated from the infected roots of field pea were *F. avenaceum*, *F. solani*, and *F. oxysporum* (Kraft, 1981; Kraft and Pfleger, 2001; Feng et al., 2010; Chittem et al., 2015; Rasiukevičiūtė et al., 2019). *Fusarium* species, especially *F. acuminatum*, have been reported to cause root rot of canola (Li et al., 2007; Chen et al., 2014).

Increasingly, *F. graminearum* has become a major problem across cereal-growing regions worldwide. For example, in Manitoba, Canada, from 1937 to 1942, *F. graminearum* was present in <0.5% of 1,448, 262, 865, and 519 samples, respectively, of wheat, durum, barley, and oats tested, compared with 16.4–39.9% for *F. poae* and 13.5–29.5% for *F. acuminatum* (Gordon, 1944). In contrast, in Saskatchewan, Canada, from 2014 to 2018, *F. graminearum* represented 23.4–55.4% (mean, 39.1% over 5 years) of all the *Fusarium* species isolated from 1,812 wheat, 71 durum, 596 barley, and 177 oat samples (Olson et al., 2019). The increased frequency or shift to *F. graminearum* has also been reported in the US, China, Brazil, Argentina, Paraguay, Uruguay, and Africa (Savary et al., 2019). Unfortunately, damage to pulse crops by *F. graminearum* has not received enough attention compared with FHB of cereals. However, the available data suggest that, among pulse crops, field pea is most susceptible to *F. graminearum* (Clarkson, 1978; Chongo et al., 2001; Goswami et al., 2008; Bilgi et al., 2011; Foroud et al., 2014; Rasiukevičiūtė et al., 2019).

In a previous study, the pea cultivar “00-2067” was found to possess partial resistance to Aphanomyces root rot, while the cultivar “Reward” was susceptible (Wu et al., 2021). In this study, we screened the cultivars “00-2067” and “Reward” to determine their reaction to five isolates representing *F. solani*, *F. avenaceum*, *F. acuminatum*, *F. proliferatum*, and *F. graminearum*. The cultivar “00-2067” was partially resistant to all five species, which suggests that it might be tolerant to many pathogens of the pea root rot complex. The difference in disease severity between the mean root rot values of the two cultivars was significant (*p* < 0.001) only for the isolates representing *F. avenaceum* and *F. graminearum*. Therefore, the F_8_ RIL population derived from “Reward” × “00-2067” was screened with F4A (*F. avenaceum*) and FG2 (*F. graminearum*) for the detection of partial resistance to the two *Fusarium* species. The greenhouse experiments were repeated four times to determine the G × E interaction for all traits. In addition, the best linear unbiased predictors (BLUPs) and LSM were applied to minimize environmental effects (Wang et al., 2018). The LSM identified six QTL, while BLUPs identified five QTL, suggesting that the LSM and BLUPs of the pooled data had comparable efficiency to detect important QTL.

Transgressive segregation was found for disease severity in the RILs inoculated with FG2 and F4A. This suggested that different resistance loci derived from the parental cultivars might have contributed to the stronger resistance observed in some of the RILs. Some transgressive RILs, such as X1303-19-3-1, X1303-21-3-1, X1303-26-2-1, X1304-21-3-1, and X1304-22-3-2, had lower disease severity in response to FG2 and higher vigor in all four environments compared with “00-2067.” In response to F4A, the RIL X1303-29-4-1 showed greater resistance and vigor compared with “00-2067.” Transgressive segregation was reported in other studies of resistance to Fusarium and Aphanomyces root rot in field pea (Feng et al., 2011; McPhee et al., 2012; Coyne et al., 2015, 2019; Nakedde et al., 2016; Wu et al., 2021). These transgressive lines will be valuable resources for developing commercial pea cultivars with improved resistance to *F. graminearum* and *F. avenaceum* and other pathogens of the pea root rot complex.

The average marker density of 1.8 marker/cM in this study was much greater than what has been reported in previous studies of pea with PCR-based markers, while the total map length (1704.9 cM) was comparable. Feng et al. (2011) constructed a linkage map of 53 cM with 14 SSR markers and obtained a marker density of 0.26 marker/cM. McPhee et al. (2012) constructed a linkage map of total length 1,716 cM with 278 PCR-based markers and reported a marker density of 0.16 marker/cm. Similarly, Coyne et al. (2015) used 178 PCR-based markers to construct a linkage map of 1,323 cM and obtained a marker density of 0.13 marker/cM. More recently, Coyne et al. (2019) have applied 914 SNP markers to construct a linkage map of total length 1,073 cM and reported a marker density of 0.85 marker/cM. A marker density of 3.5 marker/cM and total map length of 843 cM were obtained when 18 pea lines were genotyped with the same SNP array set used in this study (Desgroux et al., 2016).

In this study, 11 QTL accounting for disease severity, vigor, and plant height were identified. The major QTL for disease resistance were located on Chrom4/LGIV, while two minor QTL were detected on Chrom5/LGIII and one QTL on Chrom3/LGV. These QTL were coincident with the QTL detected for resistance to Aphanomyces root rot (Wu et al., 2021). The major QTL (*R*^2^ = 68–80%) identified by McPhee et al. (2012) for resistance to *F. oxysporum* were also located on Chrom4/LGIV, while three minor QTL (*R*^2^ = 2.8–5.4%) were located on Chrom5/LGIII. Despite identifying the same chromosomes, the similarity of the location of the QTL cannot be confirmed, given the different markers used in the two studies. However, the coincidence of the QTL is not surprising, since very few partially resistant pea cultivars are used in breeding programs across the world. Feng et al. (2011) reported that the major QTL for root rot severity caused by *F. avenaceum* were located on Chrom7/LGVII. Coyne et al. (2015, 2019) reported that the major QTL for resistance to *F. solani* were located on Chrom6/LGII, while several minor QTL were located on Chrom5/LGIII, Chrom4/LGIV, Chrom6/II, and Chrom7/LGVII.

Significant QTL × QTL interactions were found between the minor QTL for disease severity and plant height but not for vigor. An interaction of the major QTL for disease severity, vigor, and height was not observed. Wu et al. (2021) reported that the same genomic regions controlled disease severity and vigor, while plant height was a poor measure of Aphanomyces root rot severity in pea. Coyne et al. (2019) treated plant height as a direct disease-related trait. In contrast, Desgroux et al. (2016) considered plant height as an agronomic trait. The reduced epistatic interaction might be due to a reduction in the detected number of additive-effect QTL from 27 in Wu et al. (2021) to 11 in the current study.

To the best of our knowledge, no genetic studies have been carried out to determine the genomic regions associated with the partial resistance of field pea to *F. graminearum*. The use of high-density SNP markers and SSR anchor markers contributed to the construction of a fine linkage map and the identification of two stable QTL located on Chrom4/LGIV associated with partial resistance to *F. graminearum*. The identified QTL showed broad resistance to *F. graminearum*, *F. solani*, *F. avenaceum*, *F. acuminatum*, and *F. proliferatum*, as well as to *A. euteiches*. This study, together with our previous report (Wu et al., 2021), suggests that “00-2067” and the transgressive RILs with lower disease severity can be used to develop pea cultivars with improved root rot resistance.

## Data Availability Statement

Heritability of each trait, marker information, linkage information, and QTL profiles are available in the main manuscript or as Supplementary Material.

## Author Contributions

LW: inoculum preparation of *Fusarium* spp., greenhouse screening of RIL population and parents for resistance to Fusarium root rot, disease rating, measurement of vigor and plant height, phenotypic data analysis, DNA extraction, PCR, genotyping with SSR markers, and writing of the manuscript. RF-A: supervision of molecular marker work, molecular data analysis, linkage map construction, QTL mapping, and writing of the manuscript. SS: principal investigator, grant application, supervision and provision of technical support to graduate student, and revision of the manuscript. K-FC: grant application, supervision, and provision of technical support for graduate students for RIL population screening in the greenhouse. S-FH: principal investigator, grant application, supervision and provision of technical support for graduate students for RIL population screening in the greenhouse, and revision of the manuscript. All authors contributed to the article and approved the submitted version.

## Conflict of Interest

The authors declare that the research was conducted in the absence of any commercial or financial relationships that could be construed as a potential conflict of interest.

## Publisher’s Note

All claims expressed in this article are solely those of the authors and do not necessarily represent those of their affiliated organizations, or those of the publisher, the editors and the reviewers. Any product that may be evaluated in this article, or claim that may be made by its manufacturer, is not guaranteed or endorsed by the publisher.

## Footnotes

1
www.pulsedb.org/

## Supplementary Material

Supplementary Figure 1 RIL population development from the parents: ‘Reward’ and ‘00-2067’.

Supplementary Table 1 Root rot severity of the parental pea cultivars “00-2067” (partially resistant) and “Reward” (susceptible) to five *Fusarium* spp. under controlled conditions in the greenhouse.

Supplementary Table 2a ANOVA for root rot severity, vigor, and plant height using the pooled data of an RIL population of pea inoculated with *Fusarium graminearum* isolate FG2 in four greenhouse experiments.

Supplementary Table 2b ANOVA for root rot severity, vigor, and plant height using the pooled data of an RIL population of pea inoculated with *Fusarium avenaceum* isolate F4A in four greenhouse experiments.

Supplementary Table 3 Candidate gene information for the major QTL.
