# Supplementary figures and images for: Identification of Quantitative Trait Loci Associated With Partial Resistance to Fusarium Root Rot and Wilt Caused by Fusarium graminearum in Field Pea
Source: Front Plant Sci. 2022 Jan 20;12:784593. doi: 10.3389/fpls.2021.784593 (PMC8812527; doi:10.3389/fpls.2021.784593)

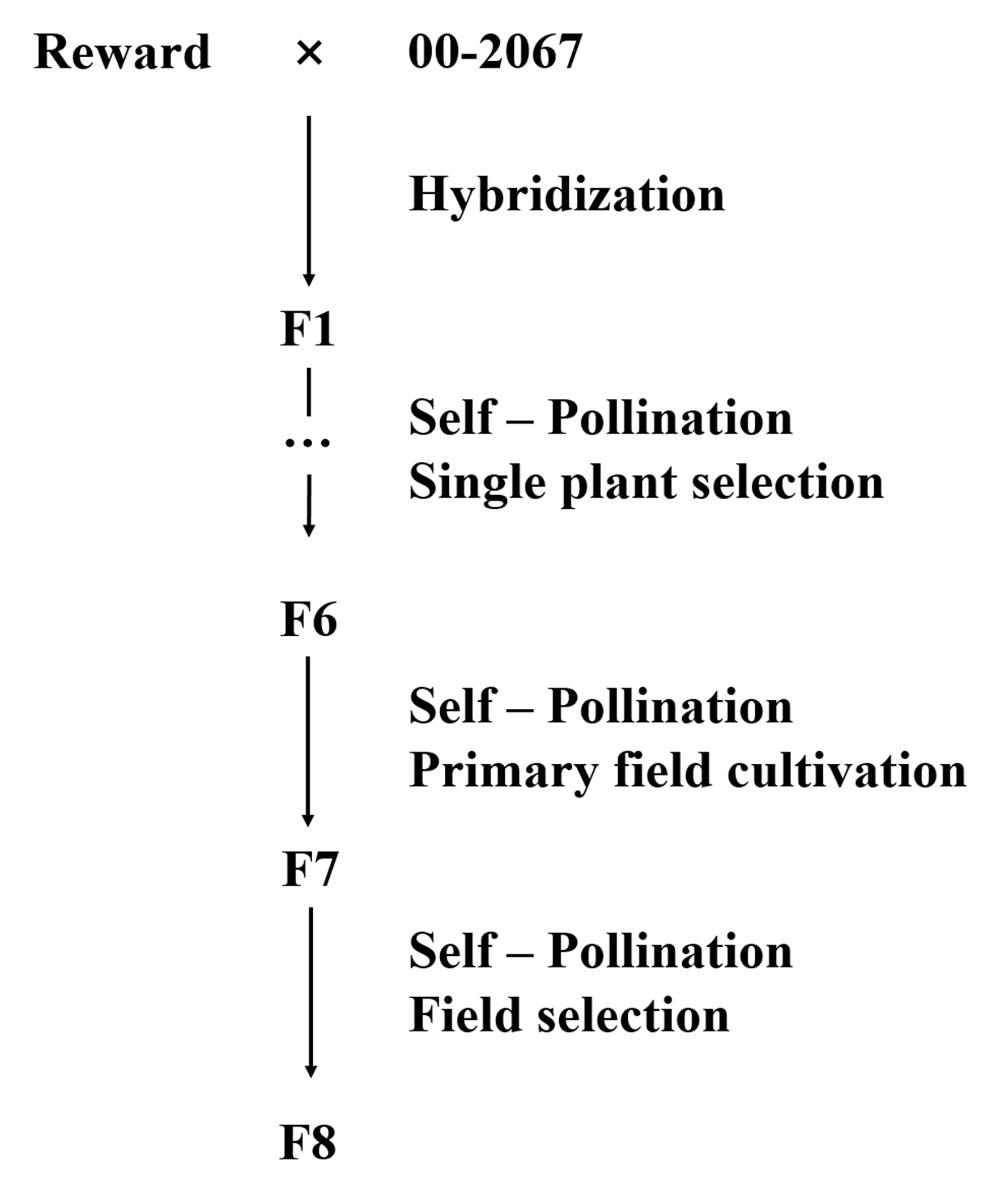

Supplement: Supplementary Figure 1 — RIL population development from the parents: ‘Reward’ and ‘00-2067’. [file Image_1.TIF]
