# Supplementary material for: Identification of Quantitative Trait Loci Associated With Partial Resistance to Fusarium Root Rot and Wilt Caused by Fusarium graminearum in Field Pea
Source: Front Plant Sci. 2022 Jan 20;12:784593. doi: 10.3389/fpls.2021.784593 (PMC8812527; doi:10.3389/fpls.2021.784593)
Supplement: Supplementary Table 1 — Root rot severity of the parental pea cultivars “00-2067” (partially resistant) and “Reward” (susceptible) to five Fusarium spp. under controlled conditions in the greenhouse. [file Table_1.docx]

**Supplementary Table 1**. Root rot severity of the parental pea cultivars ‘00-2067’ (partially resistant) and ‘Reward’ (susceptible) to five *Fusarium* spp. under controlled conditions in the greenhouse.

| Pathogen | *F. solani* | *F. avenaceum* | *F. acuminatum.* | *F. proliferatum* | *F. graminearum* |
| --- | --- | --- | --- | --- | --- |
| Isolates | **S4C** | **F4A** | **F037** | **F039** | **FG2** |
| 00-2067 | 3.1±1.2 | 1.8±0.5 | 0.8±0.2 | 2.2±0.5 | 1.1±0.4 |
| Reward | 3.7±0.3 | 2.8±0.2 | 1.8±0.6 | 3.3±0.5 | 3.3±0.4 |
| T-test | 0.1022 | 3.94E-04 | 1.122 E-03 | 0.001103 | 4.84E-07 |
