# Supplementary material for: Identification of Quantitative Trait Loci Associated With Partial Resistance to Fusarium Root Rot and Wilt Caused by Fusarium graminearum in Field Pea
Source: Front Plant Sci. 2022 Jan 20;12:784593. doi: 10.3389/fpls.2021.784593 (PMC8812527; doi:10.3389/fpls.2021.784593)
Supplement: Supplementary Table 2a — ANOVA for root rot severity, vigor, and plant height using the pooled data of an RIL population of pea inoculated with Fusarium graminearum isolate FG2 in four greenhouse experiments. [file Table_2.docx]

**Supplementary Table 2a.** Analysis of variance (ANOVA) for root rot severity, vigor and plant height using the pooled data of a RIL population of pea inoculated with *Fusarium graminearum* isolate FG2 in four greenhouse experiments.

| Source of Variance | df | Mean square | | |
| --- | --- | --- | --- | --- |
|  |  | DS | Vigor | Height |
| Genotype (G) | 128 | 4.23*** | 3.90*** | 63130*** |
| Year-station (Y-S) | 3 | 5.25*** | 17.78*** | 213909*** |
| Rep | 3 | 0.96 | 3.51** | 38462*** |
| G*Y-S | 379 | 0.32 | 0.65 | 7228 |
| Residuals | 934 | 0.57 | 0.67 | 6826 |
| Heritability |  | 0.92 | 0.86 | 0.91 |

Note: Significance difference codes: 0 ‘***’; 0.001 ‘**’; 0.01 ‘*’; 0.05 ‘.’; 0.1 ‘ ’; 1

**Supplementary Table 2b.** Analysis of variance (ANOVA) for root rot severity, vigor and plant height using the pooled data of a RIL population of pea inoculated with *Fusarium avenacium* isolate F4A in four greenhouse experiments

| Source of Variance | df | Mean square | | |
| --- | --- | --- | --- | --- |
|  |  | DS | Vigor | Height |
| Genotype (G) | 129 | 9.34*** | 12.54*** | 63149*** |
| Year-station (Y-S) | 3 | 5.39*** | 7.82*** | 91477*** |
| Rep | 3 | 9.59*** | 25.56*** | 177623*** |
| G*Y-S | 380 | 0.76* | 1.72*** | 13266*** |
| Residuals | 1327 | 0.64 | 1.21 | 8752 |
| Heritability |  | 0.92 | 0.86 | 0.79 |

Note: Significance difference codes: 0 ‘***’; 0.001 ‘**’; 0.01 ‘*’; 0.05 ‘.’; 0.1 ‘ ’; 1
